# Spontaneous Coronary Artery Dissection and Takotsubo Syndrome: Converging Mechanisms, Diagnostic Pitfalls, and a Unified Clinical Algorithm

**DOI:** 10.3390/jcm15155810

**Published:** 2026-07-24

**Authors:** Michele Golino, Khalid Shakfeh, Ashley A. Stiglich, Cristina Font, Fabiana Rollini, Madeline K. Mahowald, Gladys Velarde, Demilade Adedinsewo, Francesco Franchi, Ali Zgheib, Georges El Khoury, Jose Rivas Rios, Pasquale Mollo, Antonio Abbate, Mihail Celeski, Michele Marchetta

**Affiliations:** 1 Division of Cardiology, University of Florida College of Medicine, Jacksonville, FL 32209, USAgladys.velarde@jax.ufl.edu (G.V.); georges.elkhoury@jax.ufl.edu (G.E.K.);; 2 Department of Internal Medicine, University of Florida College of Medicine, Jacksonville, FL 32209, USA; 3 Department of Cardiovascular Medicine, Mayo Clinic, Jacksonville, FL 32224, USA; 4 Department of Cardiology, Fabrizio Spaziani Hospital, 03100 Frosinone, Italy; 5 Berne Cardiovascular Research Center, Division of Cardiology, University of Virginia, Charlottesville, VA 22908, USAmichelemarchetta00@gmail.com (M.M.); 6 Department of Medicine, Vanderbilt University Medical Center, Nashville, TN 37232, USA

**Keywords:** spontaneous coronary artery dissection, Takotsubo syndrome, myocardial infarction with non-obstructive coronary arteries (MINOCA), catecholamines, fibromuscular dysplasia, postpartum

## Abstract

Spontaneous coronary artery dissection (SCAD) and Takotsubo syndrome (TTS) are two conditions presenting as acute coronary syndromes that predominantly affect women and are frequently triggered by emotional or physical stress. While traditionally considered distinct entities, emerging evidence suggests that these conditions may coexist more frequently than previously recognized, sharing common pathophysiological mechanisms centered on catecholamine-mediated pathways, vascular vulnerability, and hormonal influences. Their overlapping presentations create significant diagnostic challenges that may lead to misdiagnosis and suboptimal management. This narrative review examines the pathophysiological convergence between SCAD and TTS, addresses the diagnostic complexity arising when these entities coexist, and proposes a novel stepwise diagnostic algorithm integrating clinical risk stratification, coronary angiography, intracoronary imaging, and cardiac magnetic resonance imaging. The review also provides scenario-dependent management guidance and outlines priorities for future research.

## 1. Introduction: The Clinical Convergence

Spontaneous coronary artery dissection (SCAD) is a non-atherosclerotic, non-traumatic, and non-iatrogenic separation of the coronary arterial wall by intramural hemorrhage, creating a false lumen that compresses the true lumen and causes myocardial ischemia. SCAD accounts for 1–4% of all acute coronary syndrome (ACS) presentations and is the leading cause of pregnancy-associated myocardial infarction, with over 90% of affected patients being women [1,2]. The mean age at presentation is 44–53 years, with a predominance in premenopausal women, though cases across all age groups have been reported [3]. Emotional stressors precede approximately 25% of SCAD events, while physical stressors (isometric exercise, Valsalva maneuvers, intense physical activity) account for an additional 30%, with no identifiable trigger in the remainder [4].

Takotsubo syndrome (TTS) is a transient form of acute myocardial dysfunction characterized by regional wall motion abnormalities that extend beyond a single coronary territory, typically in the setting of emotional or physical stress. TTS represents 1–2% of patients presenting with suspected ACS or ST-elevation myocardial infarction, with over 90% of patients being postmenopausal women (mean age: 65 years), though significant overlap in age distribution with SCAD exists [5,6]. Emotional or physical triggers are identified in approximately 65–70% of TTS cases, while no trigger is identified in 30–35% [7].

Both conditions typically present with acute chest pain mimicking ST-elevation or non-ST-elevation myocardial infarction, often prompting emergent cardiac catheterization. In SCAD, patients may present with chest pain (96%), ST-elevation (26–49%), troponin elevation (nearly universal), and occasionally with cardiogenic shock or ventricular arrhythmias, particularly in cases involving proximal or multivessel dissection [1,3]. In TTS, the most common presenting symptoms are chest pain (75%) and dyspnea (47%), with ST-elevation in approximately 44% of cases; hemodynamic complications including acute heart failure, left ventricular outflow tract obstruction, cardiogenic shock, and ventricular arrhythmias occur in up to 21.8% of patients during hospitalization [5,6].

The comorbidity profiles of SCAD and TTS differ in clinically relevant respects. SCAD patients typically have few traditional cardiovascular risk factors but a high prevalence of fibromuscular dysplasia (25–86%), connective tissue disorders, migraine, and extracoronary vascular abnormalities [1,8]. TTS patients, by contrast, have a higher prevalence of traditional cardiovascular risk factors (hypertension, diabetes, dyslipidemia), as well as psychiatric comorbidities—including anxiety disorders, depression, and prior neurological conditions—which are present in up to 55% of cases and may reflect an underlying predisposition to autonomic dysregulation. Malignancy and critical illness are recognized triggers for secondary (physical-trigger) TTS [5,6,9].

The coexistence of SCAD and TTS has been increasingly reported. In a tertiary center registry, concomitant TTS was identified in approximately 56% of patients with angiographically confirmed SCAD who underwent left ventriculography [10]. Separately, among patients with a provisional diagnosis of TTS, definite SCAD in the left anterior descending artery was present in 2.5% of cases, while an additional 7.5% had indeterminate angiographic findings for SCAD [11]. These estimates are not directly comparable: The 56% figure derives from a highly selected population of angiographically confirmed SCAD patients who underwent left ventriculography at a single tertiary center, while the 2.5% reflects the prevalence of definite SCAD among patients initially diagnosed with TTS in a separate cohort. The true prevalence of concurrent SCAD–TTS in unselected ACS populations remains undefined. This overlap may be further underrecognized due to diagnostic challenges, as both conditions can present with apical wall motion abnormalities [12].

Recognizing concurrent SCAD–TTS has important clinical implications. Although conservative management is generally preferred in both conditions, treatment must be individualized, particularly regarding antiplatelet therapy, beta-blockers, imaging follow-up, and long-term surveillance [5,6]. The rationale for examining SCAD and TTS together in this review is not to conflate two pathophysiologically distinct entities but rather to address the clinically urgent problem of their coexistence—which, if unrecognized, may lead to misdiagnosis and potentially harmful management decisions, including inappropriate revascularization, contraindicated vasoactive therapy, or premature diagnostic closure.

The aims of this narrative review are threefold: (1) to synthesize current evidence on the shared and divergent pathophysiological mechanisms linking SCAD and TTS; (2) to analyze the diagnostic pitfalls that arise when these conditions coexist and to propose a structured stepwise diagnostic algorithm; and (3) to provide scenario-dependent management guidance for concurrent SCAD–TTS.

## 2. Shared Pathophysiology

### 2.1. SCAD and TTS Within the Type 2 Myocardial Infarction Framework

Both SCAD and TTS fall within the broader spectrum of myocardial infarction with non-obstructive coronary arteries (MINOCA) and are recognized causes of, or mimics of, Type 2 myocardial infarction (T2MI), defined by the Fourth Universal Definition of MI as myocardial injury with necrosis resulting from a mismatch between oxygen supply and demand in the absence of acute atherothrombotic plaque disruption [13]. SCAD produces T2MI through mechanical luminal compromise by the intramural hematoma, while TTS—though sometimes classified separately as a non-ischemic myocardial injury—shares the supply-demand mismatch paradigm via catecholamine-mediated microvascular dysfunction and direct myocardial injury [14]. Notably, T2MI disproportionately affects women, who represent up to 55–60% of T2MI cases in contemporary registries, yet remain underrepresented in clinical trials and diagnostic algorithms [15]. Sex-specific etiologies of T2MI in women include coronary vasospasm, microvascular dysfunction, and SCAD, with hormonal influences playing a modulatory role: premenopausal estrogen exerts vasoprotective effects through nitric oxide-mediated endothelial function and attenuation of sympathoadrenal activation, while estrogen withdrawal at menopause amplifies microvascular vulnerability and catecholamine toxicity [16]. Progesterone-mediated arterial wall remodeling during pregnancy and the postpartum period further increases susceptibility to both SCAD and, potentially, stress cardiomyopathy [17]. Framing SCAD and TTS within the T2MI construct underscores that these conditions are not rare curiosities but clinically significant contributors to the sex-specific burden of acute myocardial injury and that their coexistence may represent a particularly high-risk phenotype within the MINOCA spectrum.

### 2.2. The Catecholamine Hypothesis

The catecholamine surge hypothesis represents the most recognized mechanistic link between SCAD and TTS. In TTS, emotional or physical stressors are identified in approximately 65–70% of cases [7]. Both positive and negative emotions can trigger TTS by strongly stimulating the release of catecholamines [9]. Psychological triggers include several traumatic emotional states, including bereavement, interpersonal conflict, fear, anger, anxiety, financial or occupational distress, and humiliation. However, triggers are not exclusively negative, as positive emotional experiences may also precipitate TTS [6]. When under stress, cerebral cognitive areas, especially the locus coeruleus, activate the hypothalamic–pituitary–adrenal axis, which increases the release of norepinephrine and adrenaline. It has been shown that norepinephrine concentrations in the coronary sinuses of TTS patients are higher, indicating a greater release of catecholamines from cardiac cells [18].

A state of supraphysiological adrenergic activation has been consistently documented in TTS. In a landmark investigation, plasma catecholamine levels measured on the first or second hospital day in patients with TTS were 2- to 3-fold higher than those recorded in patients with myocardial infarction and 7- to 34-fold above reference values [19]. In SCAD, catecholamines may not directly damage the coronary wall through ischemia but may act as a critical precipitating trigger. Physical or emotional stressors associated with adrenergic activation precede up to 56.5% of SCAD events, while catecholamine surges may increase coronary shear stress and trigger dissection [3,4].

#### 2.2.1. Catecholamine Effects on the Myocardium

##### The β2-Adrenergic Receptor Gs-to-Gi Signaling Switch

A key mechanism proposed to explain the apical wall motion abnormality in TTS is the β2-adrenergic receptor Gs-to-Gi signaling switch [20]. Under physiological sympathetic activation, β1- and β2-adrenergic receptors couple to the stimulatory Gs protein, activating adenylyl cyclase, increasing cyclic AMP (cAMP), and producing positive inotropy. However, when circulating epinephrine reaches supraphysiological concentrations (e.g., during acute emotional or physical stress), signaling through the β2-adrenergic receptor (β2AR) undergoes a qualitative shift: The receptor switches its Gs protein coupling to the inhibitory Gi pathway [20]. This phenomenon, “stimulus trafficking” or “biased agonism”, results in a negatively inotropic state, reversing the contractile response of cardiomyocytes to epinephrine at high concentrations [20,21]. This mechanism was subsequently validated in vivo in a rat model in which only high-dose intravenous epinephrine reproducibly generated the characteristic reversible apical myocardial depression coupled with basal hypercontractility, mimicking human TTS [21]. Importantly, β2AR density and epinephrine responsiveness were greater in apical than basal cardiomyocytes, supporting the apex-to-base gradient [21]. This Gi-coupled switch may protect the apex from calcium overload and catecholamine toxicity during acute stress, but at the cost of transient severe apical dysfunction [20,21]. However, this mechanism does not fully explain the non-apical TTS variants, suggesting additional roles for sympathetic nerve dysfunction, microvascular spasm, and central neurogenic mechanisms [22,23].

##### Regional Distribution of β-Adrenergic Receptors and Sympathetic Innervation

A fundamental anatomical basis for the apical predilection of TTS lies in the heterogeneous distribution of adrenergic receptors and sympathetic innervation across the left ventricular wall. Studies in mammalian heart models demonstrate the highest density of β-adrenergic receptors in the apical myocardium, with a decreasing gradient from apex to base [23,24]. Conversely, sympathetic nerve terminal density follows the opposite gradient. Therefore, the apex is exposed to high circulating concentrations of epinephrine while receiving little local norepinephrine release from sympathetic nerves to counteract or modulate the signal, creating the conditions for maximal β2AR–Gi-mediated cardiodepression at the apex [22,24]. Atypical patterns involving other ventricular segments may correspond to individual variation in receptor distribution and sympathetic innervation [22].

##### Direct Catecholamine Myotoxicity, Contraction Band Necrosis, and Inflammatory Response

Supraphysiological catecholamine concentrations can also exert direct toxic effects on cardiomyocytes. Through cAMP-mediated pathways and calcium overload, catecholamines induce myofibrillar hypercontraction, cellular energy depletion, and oxidative stress, leading to contraction band necrosis, characterized by hypercontracted sarcomeres and dense eosinophilic transverse bands. This finding has been consistently identified in endomyocardial biopsy specimens from TTS patients [19]. Myocardial catecholamine toxicity in TTS may result from both circulating adrenal catecholamines and local sympathetic norepinephrine spillover, producing regional stunning that often follows patterns of sympathetic innervation rather than a single coronary territory [22,23]. This adrenergic “double hit” may explain the severe transient dysfunction despite modest biomarker release. More broadly, stress cardiomyopathy has been characterized as a form of neurocardiogenic myocardial stunning associated with a non-negligible rate of serious complications, and emerging evidence implicates the NOD-like receptor family pyrin domain-containing 3 (NLRP3) inflammasome and interleukin-1 (IL-1) pathway as mediators of catecholamine-induced myocardial inflammation, representing potential future therapeutic targets [25,26].

#### 2.2.2. Catecholamine Effects on Coronary Arteries

The mechanisms by which catecholamine excess can precipitate SCAD primarily operate at the level of the coronary arterial wall and vascular tone. Three pathways have been proposed, each with a different level of supporting evidence:

(1) Shear stress amplification (inferential): The acute increase in myocardial contractility, heart rate, and arterial pressure driven by catecholamine-mediated β1- and α1-adrenergic receptor activation markedly elevates intravascular shear stress at the coronary arterial wall [3,27]. In coronary arteries with pre-existing structural vulnerability, such as those affected by fibromuscular dysplasia (FMD), this abnormal shear stress may concentrate at regions of wall weakness, precipitating either an intimal tear or spontaneous rupture of vasa vasorum, leading to intramural hematoma and luminal compression. However, no study has directly measured catecholamine levels at SCAD onset, and this mechanism remains inferential.
(2) Coronary vasospasm (supported by clinical observation): Adrenergic stimulation can induce epicardial coronary artery vasospasm, reducing vessel caliber and concentrating circumferential wall stress at the affected segment. Coronary vasospasm has been documented angiographically in SCAD patients and is a recognized trigger for both conditions [3].
(3) Exogenous sympathomimetic exposure (supported by case-level clinical data): Sympathomimetic agents, including cocaine, amphetamines, and exogenous epinephrine, have been associated with SCAD in case reports and small series [28].

A key distinction from TTS pathophysiology should be emphasized: in TTS, the catecholamine-mediated injury is primarily myocardial (receptor-mediated stunning, microvascular dysfunction, and direct myotoxicity), whereas in SCAD, the catecholamine-mediated injury is primarily vascular (shear stress, vasospasm, and arterial wall disruption in structurally predisposed vessels). The ‘two-hit’ model of SCAD—requiring both an underlying predisposed substrate and an acute precipitating trigger—is directly analogous to the proposed pathogenesis of TTS, where a pre-existing predisposition (genetic, hormonal, or neurobiological) interacts with an acute stressor to produce disease. However, the nature of this substrate differs fundamentally: In TTS, it is predominantly neurobiological and receptor-mediated, whereas in SCAD, it is structural and arteriopathic [3,28].

### 2.3. Vascular Vulnerability: Fibromuscular Dysplasia and Arteriopathy

Fibromuscular dysplasia (FMD) is a noninflammatory, nonatherosclerotic arteriopathy of small- to medium-sized arteries capable of producing stenosis, aneurysms, and dissections across multiple vascular beds [1,29]. The reported prevalence of FMD in SCAD cohorts varies considerably, ranging from 25% to 86% [8]. The wide range in reported prevalence likely reflects differences in screening completeness and imaging modality rather than true heterogeneity. Current consensus from the Canadian and European SCAD study groups recommends that all SCAD patients undergo at least one lifetime head-to-pelvis CT angiography or MRA to screen for FMD and other extracoronary arteriopathies [1,29].

FMD-affected arteries are structurally predisposed to dissection and aneurysm formation, and their close anatomical and mechanistic relationship with SCAD has led some authors to propose that SCAD may represent a coronary manifestation of systemic FMD [8,30]. SCAD patients exhibit a high burden of extracoronary vascular abnormalities beyond FMD, including abnormalities in the abdomen, pelvis, and neck [31]. Intracranial aneurysms are also found in these patients [31,32]. Cervical artery dissection in patients with FMD carries a substantial risk of stroke [32]. Coronary artery tortuosity, present in up to 78% of SCAD patients, is itself increasingly recognized as a marker of underlying arteriopathy and has been independently associated with FMD [1,33]. Tortuosity may reflect a generalized defect in arterial wall structural integrity, and its presence correlates with higher coronary tortuosity scores among patients with extracoronary FMD [33].

#### Potential Vascular Vulnerability in TTS and the Role of Microvascular Dysfunction

Coronary microvascular dysfunction (CMD) has emerged as a unifying pathophysiological substrate across the spectrum of Myocardial Infarction with Non-Obstructive Coronary Arteries (MINOCA). This finding might support the concept that SCAD and TTS are not merely co-occurring conditions but rather phenotypically distinct end-organ expressions of a common adrenergic vulnerability. Invasive studies have demonstrated elevated microvascular resistance and impaired coronary flow reserve during acute TTS, with recovery paralleling ventricular function restoration, and experimental evidence has shown that apico-basal perfusion abnormalities driven by CMD are sufficient to reproduce the TTS phenotype [34,35,36].

CMD in TTS is primarily functional, driven by catecholamine-mediated endothelial dysfunction, microvascular vasoconstriction, and autonomic dysregulation, rather than the structural arteriopathy seen in SCAD-FMD [37,38,39].

Notably, coronary tortuosity has been reported to be more frequent in TTS patients compared to matched controls, a finding that may suggest a shared predisposition toward altered arterial wall structure [40]. Genetic studies have explored polymorphisms in adrenergic receptor (ADRB1, GRK5) and estrogen receptor alpha (ESR1) genes, but no causative gene has been confirmed [41]. Postmenopausal estrogen deficiency likely amplifies catecholamine toxicity and impairs microvascular protection, helping explain the strong female and postmenopausal predominance of TTS [22]. Together, the clinical overlap and shared hormonal, autonomic, and structural substrates between SCAD and TTS suggest that a subset of patients with both conditions may harbor a systemic vulnerability that affects the macro- and microvasculature.

### 2.4. Hormonal Influences

The female predominance in both SCAD and TTS exceeds 90% [1,6]. In SCAD, estrogen withdrawal may contribute to vascular fragility by impairing endothelial integrity and collagen synthesis. The predominance of SCAD in premenopausal women, however, suggests that risk is not explained by estrogen deficiency alone. Instead, hormonal fluctuations, particularly during pregnancy and the postpartum period, may play a central role in increasing arterial wall vulnerability [1]. In the peripartum setting, progesterone weakens the arterial media by suppressing elastin expression and reorganizing collagen fibers [17], while pregnancy hormones reversibly dampen vascular smooth muscle cell traction forces, producing a mechanically vulnerable phenotype [42]. Superimposed hemodynamic stress, including plasma volume expansion of 30–50%, cardiac output peaking at 10–11 L/min during labor, and Valsalva efforts, increases coronary shear stress in this predisposed wall, explaining why peripartum SCAD clusters in the first postpartum week and presents with more severe multivessel dissections and lower LVEF than non-pregnancy SCAD [43]. In TTS, estrogen deficiency has long been proposed as a facilitating mechanism. Preclinical data demonstrate that estrogen supplementation in ovariectomized rodents attenuates catecholamine-induced myocardial stunning, likely by modulating sympathoadrenal activation and enhancing β2-adrenergic receptor signaling via the Gαi inhibitory pathway [44,45]. Estrogen also reduces the cardiovascular response to psychological stress by lowering catecholamine release and microvascular vasoconstriction [44]. However, clinical data are inconsistent: Brenner et al. found that estradiol levels were higher in TTS patients at the time of the acute event compared to women with acute MI and normal controls, reflecting a stress-induced estrogen surge rather than chronic deficiency [46]. A subsequent larger study found no significant difference in sex hormone levels between postmenopausal TTS patients and controls, nor any protective effect of hormone replacement therapy on TTS risk [47]. The current consensus is that estrogen depletion provides a biological background of susceptibility, amplifying catecholamine toxicity and reducing microvascular protection, but is unlikely to be sufficient on its own to cause TTS [45,48]. Indeed, not the absolute hormonal levels but periods of hormonal flux, including peripartum, perimenopausal, or exogenous hormonal states, may represent transient windows of heightened vascular and myocardial vulnerability.

### 2.5. The “Chicken or Egg” Dilemma

**Figure 1:**
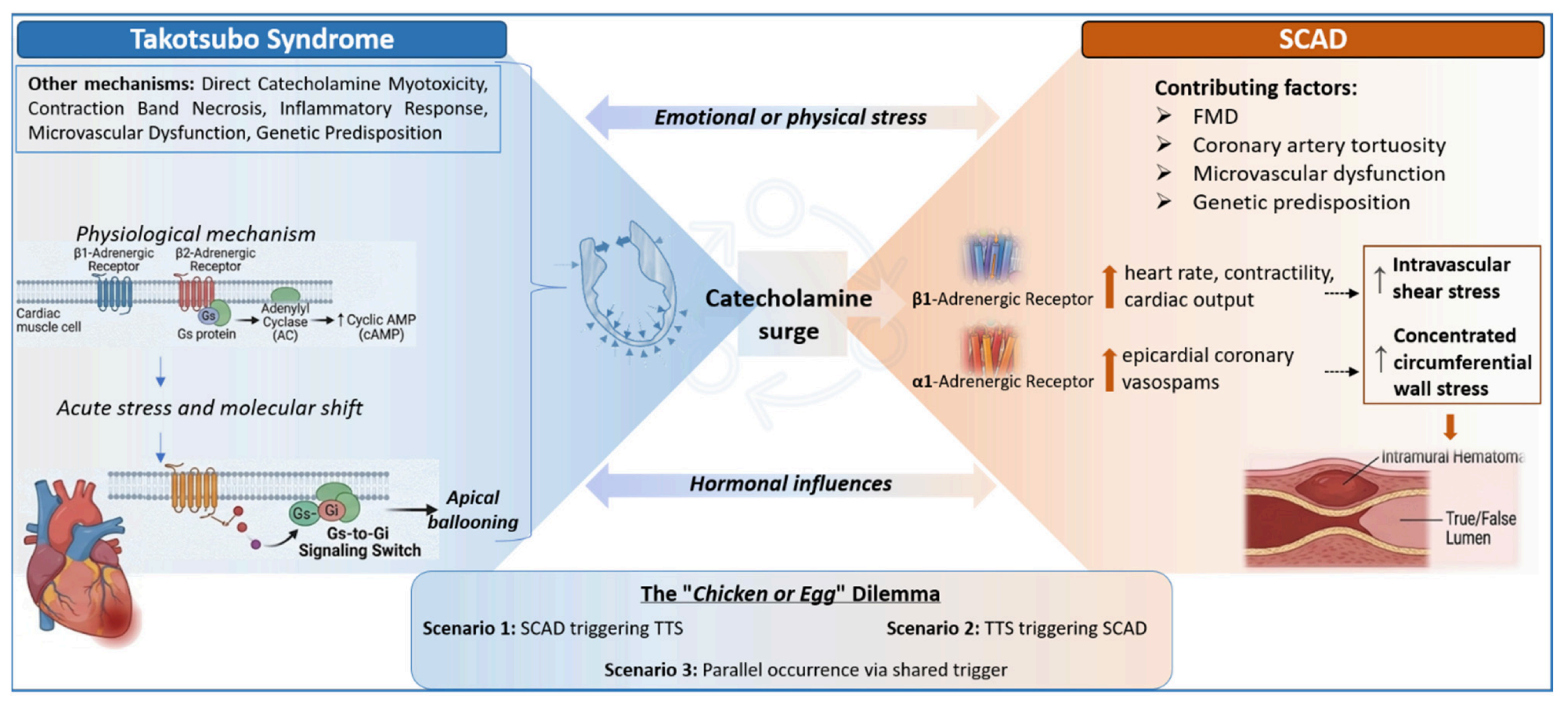
Proposed shared pathophysiological mechanisms linking spontaneous coronary artery dissection and Takotsubo syndrome. This figure summarizes the hypothesized mechanistic overlap between spontaneous coronary artery dissection (SCAD) and Takotsubo syndrome (TTS). Emotional or physical stress may trigger a catecholamine surge, which represents a central shared pathway between the two conditions. In TTS, excess catecholamine signaling may promote myocardial stunning via shifts in β-adrenergic signaling, microvascular dysfunction, direct myocyte injury, and inflammatory activation, resulting in transient ventricular dysfunction. In SCAD, the same adrenergic and hemodynamic stress may increase coronary shear stress, promote vasospasm, and contribute to arterial wall disruption in predisposed individuals, particularly those with fibromuscular dysplasia, coronary tortuosity, or underlying genetic susceptibility. Hormonal influences may further modulate vulnerability to both conditions. The figure also illustrates the unresolved relationship between SCAD and TTS, which may occur sequentially, with one condition precipitating the other, or simultaneously in response to a shared trigger. The lower panel illustrates the three proposed mechanistic scenarios: Scenario 1 (SCAD triggering TTS), Scenario 2 (TTS triggering SCAD), and Scenario 3 (parallel occurrence via a shared trigger). Abbreviations: AC, adenylyl cyclase; β1, beta-1; β2, beta-2; cAMP, cyclic adenosine monophosphate; FMD, fibromuscular dysplasia; Gi, inhibitory G protein; Gs, stimulatory G protein; SCAD, spontaneous coronary artery dissection; TTS, Takotsubo syndrome.

The temporal and causal relationship between SCAD and TTS when they occur concurrently remains unresolved. The two conditions share not only demographics and triggers but also a strikingly similar clinical presentation, making retrospective determination of which condition initiated the cascade particularly challenging [12,49]. Shared pathophysiological mechanisms between SCAD and TTS are illustrated in Figure 1. Three principal mechanistic scenarios have been proposed, and they may not be mutually exclusive [50].

#### 2.5.1. Scenario 1—SCAD Triggers TTS

Acute coronary dissection generates intense chest pain, ischemic myocardial stress, and hemodynamic instability, each of which constitutes a powerful physiological stressor potentially driving a secondary catecholamine surge. In a patient already vulnerable to adrenergic myocardial stunning, this surge may be sufficient to produce TTS as a complication of SCAD [12,49]. Supporting this sequence, Y-Hassan and colleagues documented cases in which the apical ballooning pattern extended beyond the ischemic territory expected from the dissected vessel alone, suggesting a superimposed catecholamine-mediated mechanism independent of the SCAD-related ischemia [49]. Moreover, cases have been described in which SCAD was initially misdiagnosed as TTS because of the dominant apical ballooning pattern on ventriculography, with the coronary dissection identified only on subsequent intracoronary imaging [12].

#### 2.5.2. Scenario 2—TTS Triggers SCAD

The intense adrenergic surge that initiates TTS may simultaneously act on the coronary vasculature, inducing epicardial vasospasm, elevating coronary wall shear stress, and precipitating dissection in an arterially vulnerable segment. Under this model, the coronary arterial injury is a vascular complication of the catecholamine excess that also produces myocardial stunning, with both conditions arising from the same primary stressor [49,50]. This pathway is supported by the frequent association between Valsalva-related and physical stressors and the concurrent development of TTS-like wall motion abnormalities in patients under intense stress [28], as well as by the macrovascular effects of catecholamines on coronary vascular tone [3]. TTS could also cause SCAD due to increased mechanical stress at the hinge point between hypercontracting segments and the akinetic stunned myocardium.

#### 2.5.3. Scenario 3—Parallel Occurrence via Shared Trigger

A single acute stressor may independently and simultaneously produce both SCAD and TTS through convergent adrenergic pathways, without either condition directly causing the other. Under this model, the catecholamine surge acts as a bifurcating trigger: The systemic adrenal epinephrine component drives the β2AR–Gi-mediated apical myocardial stunning of TTS, while the concurrent elevation in shear stress and coronary vasomotor instability precipitates intimal disruption or intramural hematoma in a vulnerable coronary artery [49,50]. The temporal coincidence of both conditions in the same patient would thus reflect a common upstream stressor with dual end-organ effects rather than a sequential causal chain. Distinguishing these scenarios has direct clinical and management implications. In Scenario 1 (SCAD to TTS), the SCAD component typically dictates conservative coronary management, as intramural hematomas heal spontaneously in approximately 90% of cases within 4 weeks; the superimposed TTS-related ventricular dysfunction is expected to recover within days to weeks, and the primary risk is misattributing the extensive wall motion abnormality to the SCAD territory alone and pursuing unnecessary revascularization [16]. In Scenario 2 (TTS to SCAD), the clinical imperative is twofold: first, identifying the concurrent SCAD is essential because coronary intervention in a catecholamine-stressed, structurally vulnerable vessel carries a high risk of dissection propagation; second, hemodynamic management of TTS must account for the coexisting coronary injury—catecholamine inotropes should be avoided given the risk of worsening both LVOTO and coronary shear stress; screening for dynamic LVOT obstruction should be performed before initiating beta-blockers or vasodilators, and phenylephrine is preferred for hemodynamic support when LVOTO is present [51]. In Scenario 3 (parallel occurrence), the shared trigger model reinforces the need for comprehensive multimodality imaging—including intracoronary imaging and early CMR—and a conservative-first approach, as both conditions are expected to resolve with supportive care in the majority of cases. Across all scenarios, the stepwise diagnostic and management algorithm proposed in Section 4 should be applied to guide individualized decision-making.

## 3. Diagnostic Challenges: When TTS Masks SCAD (and Vice Versa)

### 3.1. Why SCAD May Be Missed in TTS

Several compounding factors contribute to the underdiagnosis of SCAD in patients presenting with apparent TTS:

Type 2 SCAD and angiographic subtlety. The most common angiographic presentation of SCAD, Type 2, accounting for 60–70% of cases, appears as a diffuse smooth stenosis without a visible intimal flap or contrast staining [2]. Unlike Type 1, Type 2 SCAD may be easily mistaken for a normal variant, mild atherosclerosis, or even vasospasm [11].

Distal coronary location. SCAD has a predilection for mid-to-distal coronary territories, and the terminal branches of the LAD may receive insufficient angiographic scrutiny when the operator’s attention has already been directed toward the characteristic apical ballooning pattern [29,52].

Diagnostic anchoring on wall motion. When circumferential apical ballooning is identified, there may be a natural cognitive tendency to anchor on the TTS diagnosis and conclude the angiographic assessment prematurely. The traditional requirement that TTS excludes culprit coronary disease may inadvertently discourage a second, meticulous look at distal coronary anatomy for subtle Type 2 dissection [52].

Coronary tortuosity as a confounder. A key angiographic marker of SCAD, marked coronary tortuosity, is also present in TTS patients at a higher rate than matched controls, further complicating interpretation [40].

The InterTAK Diagnostic Score and the risk of premature non-invasive closure. The InterTAK Diagnostic Score, a validated seven-parameter clinical tool for distinguishing TTS from ACS, assigns a high probability to TTS when the score is ≥70 and recommends echocardiography rather than invasive angiography in this subset [53]. However, in a small real-world validation study (*n* = 10 high-scoring patients), two ultimately had SCAD rather than TTS. While limited by its very small sample size, this finding is hypothesis-generating and underscores the potential risk of premature deferral of coronary angiography in high-scoring patients, particularly when clinical features suggestive of SCAD are present [54]. In the context of SCAD–TTS overlap, the score’s variables (female sex, emotional trigger, QTc prolongation, and absence of ST depression) are present in both conditions and do not reliably separate them.

### 3.2. Why TTS May Be Missed in SCAD

Attribution of wall motion abnormalities to ischemia alone. In patients with angiographically confirmed SCAD, the WMA is often assumed to reflect the ischemic territory of the dissected vessel. However, when WMAs extend beyond the expected coronary territory, particularly when mid-to-distal LAD SCAD is associated with apical ballooning involving all segments, including those not perfused by the LAD, concomitant TTS should be considered [10].

Rapid and complete ejection fraction recovery. Early normalization of left ventricular function (within days) is the hallmark of TTS. In patients with SCAD managed conservatively, this rapid recovery may be interpreted as evidence of a “small MI” or as SCAD spontaneously healing, rather than as a separate TTS process [52].

ECG pattern misattribution. The diffuse deep T-wave inversions and marked QTc prolongation characteristic of TTS can be present in SCAD, particularly LAD SCAD. These changes are often attributed to ischemic inversion, and the possibility that they represent an overlapping TTS pattern is not considered [6].

### 3.3. Key Discriminating Features

Several clinical and imaging features can help the clinician distinguish isolated SCAD from concurrent SCAD–TTS (Table 1). The disparity between the modest troponin elevation and the extent of left ventricular dysfunction is a particularly important clinical clue for TTS and has been incorporated into formal diagnostic criteria [6]. Electrocardiographic findings deserve particular attention in the differential diagnosis. TTS characteristically follows a four-phase ECG evolution: (1) initial ST-elevation, often diffuse and involving multiple coronary territories, which may be indistinguishable from STEMI at presentation; (2) ST normalization with development of diffuse, deep, symmetric T-wave inversions, typically within 24–48 h; (3) progressive QTc prolongation, which may exceed 500 ms and carries a risk of torsades de pointes; and (4) gradual T-wave normalization over weeks to months [6,55,56]. By contrast, SCAD-related ECG changes are typically territory-specific, confined to the leads corresponding to the dissected coronary artery, and do not exhibit the diffuse, multi-territory pattern or the marked QTc prolongation characteristic of TTS. When diffuse deep T-wave inversions with QTc prolongation > 500 ms are observed in a patient with angiographically confirmed SCAD, particularly when the ECG changes extend beyond the expected territory of the dissected vessel, concurrent TTS should be actively considered. Similarly, wall motion abnormalities extending beyond a single coronary territory should prompt formal consideration of TTS as a concurrent entity rather than a bystander finding.

Notably, two-dimensional speckle-tracking echocardiography (2D-STE) can further aid in the early distinction between TTS and acute myocardial infarction. Unlike conventional echocardiography, 2D-STE differentiates between active regional myocardial contraction and passive tethered movement and can detect preserved longitudinal shortening in visually akinetic wall segments, indicating myocardial viability rather than irreversible necrosis [57,58,59,60]. In patients with severe TTS-related acute LV dysfunction, serial 2D-strain analysis revealed synergistic and synchronous longitudinal strain and strain-rate patterns even during acute episodes, despite the akinetic appearance of the LV apex, while end-systolic circumferential wall stress was several times higher in apical than basal regions, suggesting that apical akinesia may partly reflect LV geometry-induced wall stress differences rather than severely impaired contractility [57,59]. These findings carry implications for the management of TTS in the intensive care setting, as the persistence of higher apical systolic wall stress was observed during catecholamine therapy and its reduction after catecholamine withdrawal, suggesting that catecholamine administration may perpetuate the ballooning phenotype. Transthoracic 2D-STE can therefore be useful for early distinction between TTS and acute apical MI when attention is focused on regional myocardial function and may provide additional diagnostic value in suspected concurrent SCAD–TTS by identifying preserved myocardial contractility and a TTS-pattern strain abnormality that extends beyond the SCAD territory.

**Table 1:** Comparison of non-invasive clinical features between SCAD and TTS.

Feature	Isolated SCAD	Isolated TTS	Concurrent SCAD–TTS
Demographics	~90% women; median age 47–53 years; low prevalence of traditional CV risk factors [1,2]	>90% women; typically postmenopausal (median ~74 years); higher prevalence of CV risk factors (hypertension, diabetes, dyslipidemia) [5,6]	Older (median ~67–74 years); higher prevalence of CV risk factors; demographic profile closer to TTS than isolated SCAD [10,61]
Precipitating triggers	Emotional stress (~25%); physical stressors (isometric exercise, Valsalva) also frequent; overall triggers in ~42% [3]	Emotional or physical triggers in ~56–70% [7]; no identifiable trigger in ~30–35% [6]	Overall triggers more frequent; physical and emotional triggers both represented [10]
Presenting symptoms	Acute chest pain (~96%); dyspnea less common; cardiogenic shock rare unless proximal/multivessel dissection present [1,2]	Chest pain (~75%); dyspnea (~47%); syncope or presyncope may occur; hemodynamic instability in up to 21.8% [5]	Chest pain predominant; hemodynamic instability may reflect both ischemic and catecholamine-mediated components [12]
Comorbidity profile	Low prevalence of traditional CV risk factors; high prevalence of FMD (25–86%), migraine, connective tissue disorders, and extracoronary vascular abnormalities [1,8]	Higher prevalence of hypertension, diabetes, dyslipidemia; psychiatric comorbidities (anxiety, depression) in up to 55%; malignancy and critical illness as triggers for secondary TTS [5,9]	Mixed profile; FMD screening warranted regardless of CV risk factor burden [1,29]
WMA pattern	Corresponds to SCAD territory; LVEF < 50% in only ~12% of patients [2]	Extends beyond a single coronary territory in a circumferential pattern: apical (82%), mid-ventricular (14.6%), basal (2.2%), and focal (1.5%) [5]	Extends beyond SCAD territory; lower LVEF and a higher WMAI score [10]
LV ballooning pattern	Regional (matches dissected artery territory) [2]	Circumferential apical ballooning most common; mid-ventricular, basal, or focal variants also possible; transitions between types can exist [6]	Circumferential apical ballooning or other segments in atypical TTS forms; WMA disproportionate to SCAD territory [10]
RV involvement	Rare (unless RCA dissection) [3]	Present in ~11% (biventricular ballooning); associated with physical triggers, higher cardiogenic shock rate, and worse long-term mortality (29% vs. 12.8%) [62,63]	May occur; clinical significance requires further characterization [12]
LVEF recovery timeline	Weeks to months; ~85.6% have WMA at baseline but 74% have LVEF ≥ 50%; WMA resolution in ~49% on follow-up; high troponin (>50 µg/L) predicts persistent dysfunction [2]	Hours to weeks, often rapid and complete; recovery typically within 2–4 weeks; 10–15% may have persistent diastolic dysfunction [6,64]	Dual trajectory: rapid recovery of TTS-related stunning, slower recovery if SCAD-related infarction is present [10,12]
ECG	Territory-specific ST changes (ST-elevation in ~50% of STEMI-SCAD) [2]; QTc usually normal or mildly prolonged [3]	Dynamic four-phase evolution: (1) diffuse ST-elevation (44%, widespread beyond single coronary territory); (2) ST normalization with progressive deep symmetric T-wave inversions (slope −43.1 vs. −16.6 mm in MI, *p* = 0.02); (3) marked QTc prolongation (>500 ms in ~64% at 24–48 h; nearly universal within 96 h); (4) gradual normalization. QTc > 500 ms is a key discriminator from MI [55,56,65]	Hybrid pattern: territory-specific changes from SCAD overlaid with diffuse T-wave inversions and dynamic QTc prolongation suggestive of TTS component [6]
Troponin/WMA disparity	Proportional to infarct size; peak troponin correlates with extent of myocardial necrosis [3]	Disproportionately low troponin relative to the extent of LV dysfunction; >95% have elevated troponin but peak values are modest relative to WMA territory [5,6]	Disproportionately low relative to the extent of dysfunction; modest troponin peak with extensive WMA suggests TTS component [6]
2D-STE strain pattern	Strain reduction confined to the territory of the dissected coronary artery, consistent with ischemic injury	Circumferential strain reduction extending beyond a single coronary territory; preserved longitudinal shortening in visually akinetic segments indicating myocardial viability rather than irreversible necrosis [57,58]	Hybrid pattern: territory-specific strain reduction in SCAD territory with circumferential strain abnormality and preserved longitudinal shortening in remote segments, suggesting superimposed TTS component [57,58]
BNP/NT-proBNP	Mildly elevated, proportional to infarct size [3]	Markedly elevated and disproportionate to troponin rise; BNP/troponin ratio often higher; BNP/TnI ratio at admission > 329 distinguishes TTS from NSTEMI; NT-proBNP/TnT ratio at peak > 2889 distinguishes TTS from STEMI (91% sensitivity, 95% specificity) [66,67]	Higher than expected for the degree of SCAD-related myocardial necrosis; elevated BNP/troponin ratio should raise suspicion for TTS component [6]
Coronary angiography	Type 1 (intimal flap/dual lumen), Type 2 (diffuse smooth stenosis, 60–70%), or Type 3 (focal, mimics atherosclerosis); mid-to-distal LAD predilection; post-nitroglycerin persistence distinguishes it from vasospasm [2,68]	No culprit obstructive lesion; epicardial coronary arteries typically normal or with mild non-obstructive atherosclerosis [6]	SCAD lesion identified + WMA extending beyond the expected territory of the dissected vessel; left ventriculography essential to document ballooning pattern [10,11]
Intracoronary imaging (OCT/IVUS)	Intramural hematoma, double-lumen morphology, intima-medial membrane separation; OCT preferred for distal/Type 3; IVUS for proximal/Type 1 [29,69]	Normal coronary wall architecture; no intramural hematoma or dissection [6]	SCAD features in dissected segment; normal wall architecture in non-dissected vessels [3]
CMR LGE	Subendocardial-to-transmural LGE strictly confined to the dissected coronary territory; ~40% of SCAD events resolve without detectable LGE [29,70]	Absent or minimal in most cases; when present (up to ~16%), LGE is less bright than MI (signal intensity < 5 SD), with concentric transmural extension; prevalence decreases with later CMR timing [25,71,72]	Ischemic LGE in SCAD territory plus absent/minimal LGE in remote dysfunctional segments; “hybrid” pattern supports concurrent diagnosis [10,73]
CMR T2-weighted edema	Localized to SCAD territory (if present) [70]	Diffuse myocardial edema extending beyond a single coronary territory; edema matches WMA distribution; T2-mapping Z-score decreases by ~0.22 units/day [71]	Diffuse myocardial edema extending beyond coronary territory; mixed pattern with ischemic LGE + diffuse edema is confirmatory [73]
Acute complications	Low in-hospital MACE (~4.7%); CHF rare (~0.6%); AF rare (~1%) [2]	Higher acute complication rate; acute HF, LVOTO, cardiogenic shock, and ventricular arrhythmias may occur; in-hospital complications in 21.8% in the International TTS Registry [5]	Higher in-hospital MACE (~12%); CHF (~10%); AF (~11%); LVOT obstruction possible [10]
In-hospital mortality	Low (~1.3%) [2]	4–5% in large TTS cohorts [5]	Higher (~4–5.7%) [10]
Diagnostic pitfall	Distal LAD SCAD (type 2b) can mimic apical TTS on ventriculography [11]	TTS may be incorrectly diagnosed when subtle distal LAD SCAD is missed on angiography [12]	Up to 2.5% of provisional TTS diagnoses may harbor missed SCAD; additional 9% are indeterminate [11]

Abbreviations: AF: atrial fibrillation; BNP: B-type natriuretic peptide; CHF: congestive heart failure; CMR: cardiac magnetic resonance imaging; CV: cardiovascular; ECG: electrocardiogram; FMD: fibromuscular dysplasia; IVUS: intravascular ultrasound; LAD: left anterior descending artery; LGE: late gadolinium enhancement; LV: left ventricle (left ventricular); LVEF: left ventricular ejection fraction; LVOT: left ventricular outflow tract; LVOTO: left ventricular outflow tract obstruction; MACE: major adverse cardiovascular events; NT-proBNP: N-terminal pro-B-type natriuretic peptide; OCT: optical coherence tomography; QTc: corrected QT interval; RCA: right coronary artery; RV: right ventricle (right ventricular); SCAD: spontaneous coronary artery dissection; TnI: troponin I; TnT: troponin T; TTS: Takotsubo syndrome; WMA: wall motion abnormality; WMAI: wall motion abnormality index.

### 3.4. Role of Intracoronary Imaging

When angiography is inconclusive, particularly in suspected Type 2 or Type 3 SCAD, intracoronary imaging with OCT or IVUS is the definitive next step. OCT provides near-histological detail of the arterial wall, identifying the intramural hematoma, double-lumen morphology, intima-medial membrane separation, and entry point when present [2,69]. IVUS offers greater penetration depth and may be preferred for larger proximal vessels and when a Type 1 false lumen is suspected [69]. The 2023 ESC/ACS guidelines now recommend OCT/IVUS for identifying culprit lesions in angiographically ambiguous cases [74]. The ESC/ACCA SCAD position paper similarly supports intracoronary imaging whenever angiographic diagnosis is uncertain [29]. In SCAD, intracoronary imaging requires specific procedural caution, as catheter-induced propagation of the intramural hematoma is a recognized complication when excessive contrast force or deep catheter engagement is used [3,29,75].

### 3.5. Role of Cardiac Magnetic Resonance Imaging

CMR is pivotal when invasive coronary angiography fails to identify an obstructive lesion or anatomical explanation for myocardial injury [76]. When performed within 7–14 days of the acute event, the diagnostic yield increases to approximately 84%; this window balances the need to capture transient myocardial edema and hyperemia before resolution against the practical constraints of acute clinical stabilization [70]. The late gadolinium enhancement (LGE) pattern is the pivotal discriminator. In SCAD-related MI, LGE follows a subendocardial-to-transmural distribution strictly confined to the relevant coronary territory and is indistinguishable from atherosclerotic MI. In TTS, however, the classical finding is diffuse myocardial edema on T2-weighted imaging without significant LGE, reflecting myocardial stunning rather than irreversible necrosis [76,77]. Notably, up to 40% of TTS patients may show LGE in the acute phase, although with lower signal intensity compared with myocardial infarction [25]. Approximately 40% of SCAD events resolve without any detectable LGE on follow-up. This overlap can create diagnostic ambiguity between SCAD and TTS [29]. In suspected concurrent SCAD–TTS, CMR may show a mixed pattern, with subendocardial LGE confined to the dissected artery’s territory and myocardial edema extending beyond that region, supporting coexisting TTS-related stunning. Serial CMR at 3–6 months is recommended to confirm functional recovery and to reassess LGE distribution as definitive evidence of the underlying mechanism [76]. The combination of OCT and CMR has been shown to identify the underlying mechanism of MINOCA in the majority of cases, with the two modalities providing complementary information [73].

## 4. Proposed Diagnostic Algorithm

**Figure 2:**
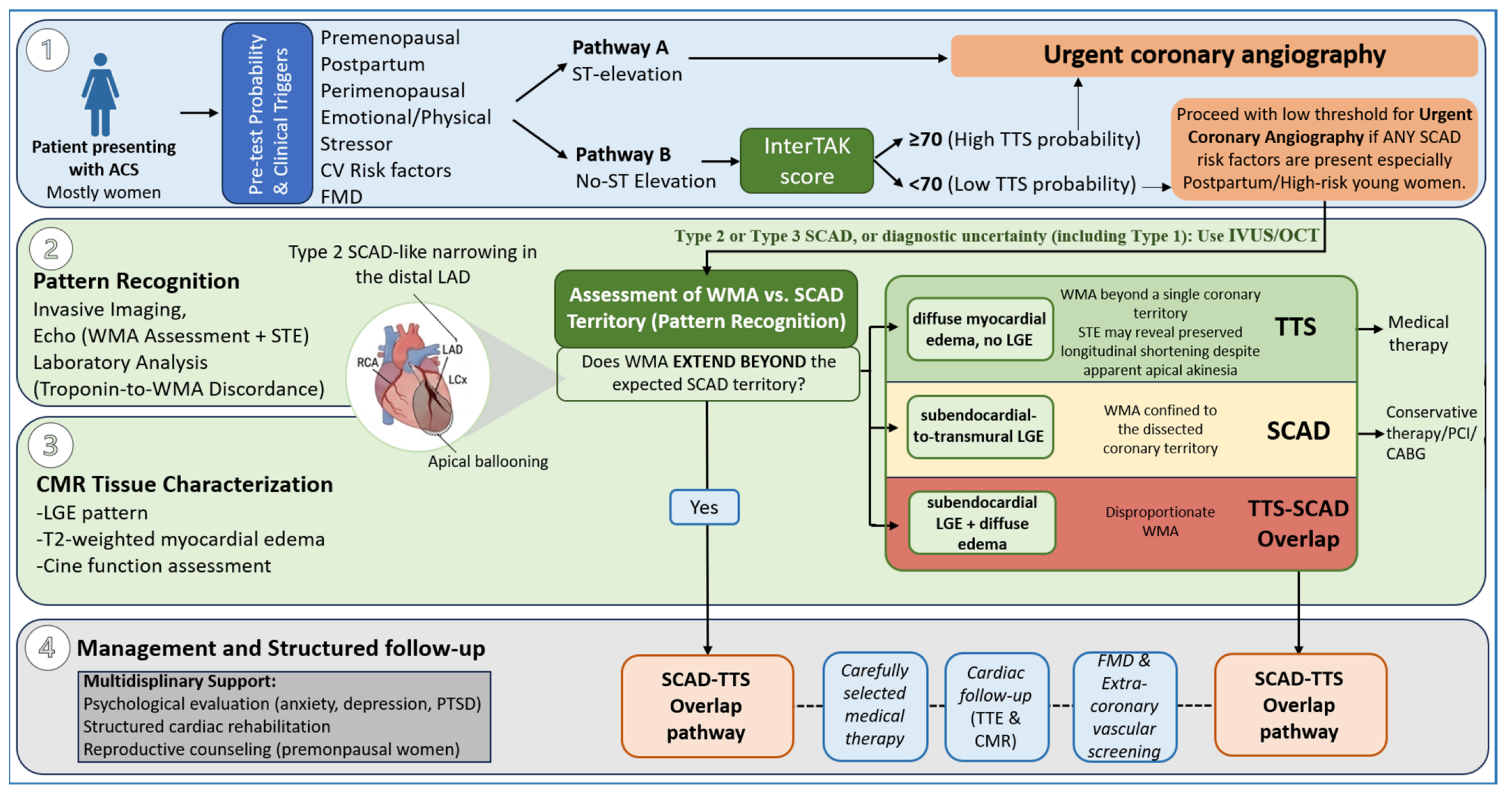
Proposed diagnostic algorithm for suspected Takotsubo syndrome–spontaneous coronary artery dissection overlap. This figure outlines a stepwise diagnostic framework for patients with suspected overlap between Takotsubo syndrome (TTS) and spontaneous coronary artery dissection (SCAD), particularly for women presenting with acute coronary syndrome. The initial evaluation incorporates clinical risk factors and electrocardiographic findings to guide the urgency of coronary angiography, with a low threshold for invasive evaluation when SCAD risk features are present. Coronary angiography, supplemented by intracoronary imaging when needed, is followed by assessment of regional wall motion abnormalities (WMA) relative to the suspected coronary territory using transthoracic echocardiography. Two-dimensional speckle-tracking echocardiography (STE) may provide incremental diagnostic value by identifying preserved longitudinal myocardial deformation despite apparent wall-motion abnormalities, thereby supporting myocardial viability and improving differentiation between TTS and acute myocardial infarction. Cardiac magnetic resonance (CMR) imaging performed within 7–14 days helps differentiate isolated TTS, isolated SCAD, and overlapping phenotypes based on the presence and distribution of myocardial edema and late gadolinium enhancement (LGE). The final step emphasizes structured longitudinal follow-up, including individualized medical therapy, repeat cardiac imaging, screening for extracoronary arteriopathies, and multidisciplinary supportive care. Abbreviations: ACS, acute coronary syndrome; CABG, coronary artery bypass grafting; CMR, cardiac magnetic resonance; FMD, fibromuscular dysplasia; IVUS, intravascular ultrasound; LAD, left anterior descending artery; LCX, left circumflex artery; LGE, late gadolinium enhancement; OCT, optical coherence tomography; PCI, percutaneous coronary intervention; PTSD, post-traumatic stress disorder; RCA, right coronary artery; SCAD, spontaneous coronary artery dissection; STE, speckle-tracking echocardiography; TTE, transthoracic echocardiography; TTS, Takotsubo syndrome; WMA, wall motion abnormality.

Based on current evidence and published guidelines, we propose a structured stepwise diagnostic algorithm for evaluating suspected concurrent SCAD–TTS in patients presenting with ACS (Figure 2).

### 4.1. LEVEL 1—Initial Triage: Who Deserves Urgent Angiography?

All women presenting with ACS should be evaluated for clinical features that raise the pre-test probability of SCAD, TTS, or their coexistence. High-index clinical triggers include premenopausal status, postpartum presentation (especially within 6 weeks of delivery), perimenopausal state, multiparity, an emotional or intense physical stressor, few traditional cardiovascular risk factors, and known or suspected FMD [1,11]. For patients presenting with ST-elevation, the 2025 American College of Cardiology (ACC)/American Heart Association (AHA) and 2023 European Society of Cardiology (ESC) guidelines recommend urgent coronary angiography regardless of suspected etiology [74,78]. For patients without ST-elevation, the InterTAK Diagnostic Score can be used as a pre-test probability tool: a score ≥ 70 indicates a high probability of TTS and may support initial echocardiographic triage. However, the threshold for proceeding to invasive angiography should remain low in women with SCAD risk factors, regardless of InterTAK score [54]. Coronary angiography, when performed, should include meticulous slow-frame review of the mid-to-distal LAD for diffuse smooth narrowing (Type 2 SCAD) after intracoronary nitroglycerin administration, which distinguishes persistent SCAD stenosis from vasospasm. Simultaneous left ventriculography is strongly recommended to document wall motion abnormalities in relation to SCAD territory [1,3].

### 4.2. LEVEL 2—Pattern Recognition: Angiographic, Echocardiographic, and Laboratory Analysis

The Yip–Saw classification was developed to aid diagnostic pattern recognition of SCAD. It categorizes angiographic features into three types: Type 1 (a dual-lumen appearance caused by contrast entering the false lumen), Type 2 (a long, smooth stenosis), and Type 3 (a lesion resembling focal atherosclerotic disease) [68]. Once SCAD is confirmed or suspected, the extent of left ventricular WMA relative to the dissected coronary territory becomes the central decision node. If WMA matches the SCAD territory, the picture is more consistent with isolated SCAD. If the diagnosis of SCAD is confirmed, subsequent management should follow the principles outlined in Section 5, with conservative therapy as the most common and generally preferred initial approach in clinically stable patients.

Because SCAD has a predilection for more distal coronary territories, including the LAD, apical regional wall motion abnormalities akin to those seen in TTS are common. Thus, if WMA extends beyond the expected SCAD territory, particularly as circumferential apical ballooning involving segments remote from the dissected vessel, concurrent TTS should be actively suspected. Left ventriculography patterns showing basal hyperkinesis with apical ballooning are characteristic of TTS and, when present alongside angiographic SCAD, should prompt urgent further evaluation [10,52]. Two-dimensional speckle-tracking echocardiography (2D-STE) may complement conventional echocardiographic assessment by detecting preserved longitudinal shortening in visually akinetic segments and identifying circumferential strain patterns extending beyond the SCAD territory, further supporting a superimposed TTS component [57,58,60].

If angiographic findings are indeterminate, intracoronary imaging with OCT (preferred for Type 3 and distal vessels) or IVUS (preferred for proximal large vessels and Type 1 suspicion) should be performed [3,29].

The troponin-to-WMA disparity is also a useful bedside signal: a disproportionately modest troponin elevation relative to the extent of ventricular dysfunction should raise suspicion for an overlapping TTS component, as extensive WMA in TTS reflects myocardial stunning rather than necrosis [52].

### 4.3. LEVEL 3—CMR Confirmation: Tissue Characterization

CMR should be performed within 7–14 days of the acute event in all patients with confirmed or suspected SCAD–TTS overlap and in all MINOCA presentations in which the angiographic diagnosis remains uncertain. The recommended CMR protocol includes cine imaging for regional and global function, LGE sequences, and T2-weighted or T1/T2 mapping sequences for myocardial edema [76]. The LGE pattern is the pivotal tissue discriminator:

Ischemic (SCAD) pattern: Subendocardial-to-transmural LGE strictly confined to the dissected coronary territory. This finding confirms MI attributable to SCAD and should prompt reassessment of angiographic images and consideration of intracoronary imaging [73,76].

TTS pattern: Diffuse myocardial edema on T2-weighted imaging, without significant or with minimal transient LGE. Regional dysfunction extending beyond a single coronary territory, combined with absent fibrosis, is strongly consistent with TTS [73].

Mixed (concurrent SCAD–TTS) pattern: Subendocardial LGE confined to the SCAD territory, accompanied by diffuse edema in segments beyond that territory. This “hybrid” CMR signature should be regarded as confirmatory of concurrent SCAD–TTS [10,73]. Absence of LGE alone cannot exclude SCAD [29].

### 4.4. LEVEL 4—Long-Term Follow-Up, Screening, and Counseling

All patients with confirmed SCAD, regardless of TTS coexistence, should enter a structured follow-up pathway:

Cardiac follow-up: c Follow-up CMR at 3–6 months may be considered to confirm functional recovery and reassess tissue characterization [3].

FMD and extracoronary vascular screening: Head-to-pelvis CT angiography or MRA is recommended for all SCAD patients at least once in their lifetime to screen for extracoronary FMD, cervical artery dissection, and intracranial aneurysms [1,79].

Medical therapy: Conservative management is the preferred strategy for hemodynamically stable SCAD patients without ongoing ischemia, left main coronary artery involvement, or cardiogenic shock [80]. Beta-blockers are widely prescribed for SCAD and are associated with reduced recurrence in observational data. For confirmed TTS without residual SCAD ischemia, ACE inhibitors or ARBs are preferred over antiplatelet agents, while beta-blockers are continued with caution, given the risk of bradycardia and pause-dependent torsades de pointes in the context of QTc prolongation [52,80]. When both conditions coexist, a hybrid approach with a beta-blocker, low-dose aspirin, and an ACE inhibitor/ARB is reasonable.

Psychological support and cardiac rehabilitation: Both SCAD and TTS carry a substantial psychosocial burden, including anxiety, depression, and post-traumatic stress, and require structured cardiac rehabilitation and “psychocardiological” evaluation [1,52].

Reproductive counseling: For premenopausal women, future pregnancy counseling should be individualized and conducted by a multidisciplinary pregnancy heart team, given the risks associated with pregnancy-related SCAD and the evidence that subsequent pregnancies carry a non-negligible recurrence risk [1,11].

## 5. Management Considerations

Management of concurrent SCAD–TTS requires an individualized approach. The shared preference for conservative management provides a unifying framework, but several specific considerations must be balanced when both diagnoses coexist [1,52,80].

### 5.1. Revascularization Strategy

Conservative management is the established first-line approach for both conditions. For SCAD, observational data consistently demonstrate that PCI is associated with high technical failure rates and worse outcomes compared to conservative therapy. In a recent SCAD registry, PCI was successful in only 34.7% of attempted cases, with in-hospital MACE rates of 29.3% in the PCI group versus 2.8% in the conservatively managed group [81]. Multiple other studies confirmed no significant advantage of PCI over conservative management for long-term MACE, recurrent MI, or heart failure outcomes [82,83]. The mechanistic rationale supports a conservative approach, as the intramural hematomas resolve spontaneously in the vast majority of cases, with angiographic healing demonstrated in approximately 90% of patients within 4 weeks, rendering stenting biologically unnecessary and mechanically hazardous [29,80]. CABG should be reserved for left main dissection, extensive proximal multivessel SCAD, or refractory ischemia not amenable to conservative management, with the caveat that long-term graft patency is poor (approximately 27% in some series) due to healing of the native vessel competing with graft flow [80]. For TTS, revascularization is not usually indicated. However, CAD and TTS may coexist [50]. Management is supportive, targeting hemodynamic stabilization and prevention of complications, including LV outflow tract obstruction, cardiogenic shock, and arrhythmias. For concurrent SCAD–TTS, the principle of conservative management is therefore doubly reinforced. Intervention should be considered only when high-risk features are present: ongoing ischemia with TIMI <2 flow, hemodynamic instability or cardiogenic shock, left main coronary artery dissection, or life-threatening ventricular arrhythmias. The AHA Scientific Statement recommends a minimum 48 h admission for SCAD, with 3–5 days justified given the 5–10% early hazard of dissection extension [1]. For TTS, the International Takotsubo Registry reported serious in-hospital complications in 21.8% of patients and 5% in-hospital mortality among patients [5]. When SCAD and TTS coexist, a monitoring period of at least 3–5 days, individualized to clinical trajectory, LVEF, and arrhythmic burden, is reasonable.

### 5.2. Medical Therapy

Table 2 summarizes current evidence-based recommendations for medical management of isolated SCAD, isolated TTS, and concurrent SCAD–TTS.

Beta-blockers require caution in acute TTS, particularly when LV outflow tract obstruction (LVOTO) is present. In this setting, which occurs in approximately 10–25% of TTS cases, beta-blockade may worsen hemodynamics by reducing compensatory sympathetic support. Phenylephrine (a pure alpha-agonist) is preferred for hemodynamic support when LVOTO is present [52]. Once LVOTO has resolved, beta-blockers may be introduced when clinically appropriate, particularly for SCAD-specific indications [80]. The BA-SCAD randomized trial is currently evaluating the optimal use of beta-blockers and antiplatelet therapy duration in SCAD [84]. Additionally, SCAD is more commonly linked to hypertension that necessitates nitrate therapy. However, nitroglycerin administration in the presence of LVOTO has been shown to exacerbate the pressure gradient in TTS and should be avoided. Additionally, patients whose ACS is complicated by cardiogenic shock may benefit from the use of catecholaminergic medications. However, it is recommended to avoid catecholamine inotropic medications when there is concurrent TTS, especially with LVOTO, as they have been associated with a 20% greater risk of death. Levosimendan, a calcium sensitizer, has been suggested as a safe and effective alternative to catecholamine medications in complex TTS, but it is not available in the United States [50].

### 5.3. Long-Term Follow-Up

Prospective data suggest that long-term outcomes after SCAD may be more favorable than previously reported, with a 0.8% mortality rate and a relatively modest recurrence rate (2.4%) [85]. Adverse events appear to be more common in patients with peripartum SCAD, underlying genetic disorders, or extracoronary FMD [85]. The optimal long-term medical strategy remains uncertain and requires further prospective validation [85].

TTS recurrence rates average approximately 1.8–5.0% annually, with pooled data from the International TTS Registry suggesting a recurrence rate of approximately 3.5% per year and a cumulative risk of up to 10% at 5 years [52]. Importantly, TTS carries hospital morbidity rates comparable to those of acute MI, with 10-year all-cause mortality and recurrent major cardiovascular events approaching those of atherosclerotic ACS [86].

## 6. Future Directions

Despite growing recognition of SCAD and TTS individually, the concurrent SCAD–TTS phenotype remains largely unstudied in prospective, dedicated registries. Multicenter prospective registries that apply both the ESC TTS diagnostic criteria and a standardized SCAD angiographic classification protocol, with routine intracoronary imaging and early CMR, would provide the epidemiological and clinical data necessary to properly characterize this overlap population, define recurrence risk, and optimize management. Major recent advances have delineated the genetic architecture of SCAD with increasing precision. The landmark 2023 genome-wide association meta-analysis identified 16 risk loci for SCAD, implicating genes involved in extracellular matrix biology, vascular smooth muscle integrity, and tissue-mediated coagulation [87]. The polygenic risk score for SCAD was associated with higher SCAD risk in FMD carriers while being inversely associated with atherosclerotic CAD and MI risk in the UK Biobank and Million Veteran Program, establishing SCAD and atherosclerotic MI as opposite ends of a single genetic risk spectrum [88]. By contrast, the genetics of TTS remain poorly characterized. Candidate gene studies of adrenergic receptor polymorphisms (ADRB1, GRK5) and estrogen receptor alpha (ESR1) have yielded inconsistent results, and no replicable common variant has been confirmed [86]. Whether SCAD and TTS share genetic susceptibility at any locus represents a compelling but entirely unexplored research question. The role of hormone replacement therapy (HRT) following either SCAD or TTS remains unanswered. In postmenopausal women with TTS, the evidence neither supports nor refutes a protective effect of exogenous estrogen on recurrence risk [52]. In SCAD, HRT counseling is addressed in only 7–85% of outpatient visits, depending on whether care is delivered in a SCAD specialist clinic versus general cardiology, suggesting a major gap in clinical practice [89].

An unexplored area concerns transgender individuals receiving gender-affirming hormone therapy (GAHT). In a large Dutch cohort, transmasculine individuals on testosterone had a significantly elevated MI risk (SIR 4.20; 95% CI 2.72–6.01) compared with cisgender women [90], potentially mediated by testosterone-induced endothelial dysfunction and vascular inflammation [91]. No case series has examined SCAD or TTS specifically in transgender and gender-diverse individuals. Future registries should systematically collect data on gender identity and GAHT to characterize cardiovascular risk in transgender and gender-diverse individuals [92].

The role of myocardial inflammation in TTS is increasingly recognized, with evidence of macrophage infiltration and elevated IL-6 persisting for months after the acute event [64]. IL-1 pathway blockade, which has shown benefit in acute myocardial infarction and is now approved for recurrent pericarditis, represents an unexplored but mechanistically plausible therapeutic strategy for TTS [93,94].

### 6.1. Limitations

This narrative review has several limitations. The literature search was not conducted according to a systematic protocol, and publication bias cannot be excluded. Evidence for concurrent SCAD–TTS derives from case reports and small registries, with no prospective studies. The proposed diagnostic algorithm has not been prospectively validated. Management recommendations are extrapolated from isolated SCAD and TTS data, as no randomized trial has addressed the overlap phenotype. The review is limited to English-language publications.

### 6.2. Contribution to Literature

This review offers several original contributions: (1) the most comprehensive pathophysiological synthesis of SCAD–TTS overlap to date, framed within the T2MI/MINOCA spectrum; (2) the first structured, stepwise diagnostic algorithm for suspected concurrent SCAD–TTS (Figure 2); (3) a scenario-dependent management framework addressing therapeutic implications based on the causal sequence; and (4) identification of underrecognized at-risk populations and novel therapeutic targets, including IL-1 pathway blockade.

## 7. Conclusions

SCAD and TTS share demographic, clinical, and pathophysiological features, including female predominance, frequent emotional or physical triggers, catecholamine excess, hormonal influences, and vascular vulnerability. These similarities support a potential mechanistic link and suggest that concurrent SCAD–TTS may be underrecognized. This review provides three clinically actionable contributions: (1) a comprehensive synthesis of the shared and divergent pathophysiology of SCAD and TTS, framed within the broader T2MI/MINOCA spectrum; (2) a structured, stepwise diagnostic algorithm (Figure 2) integrating clinical risk stratification, coronary angiography with intracoronary imaging, wall motion territory analysis, and CMR tissue characterization to identify concurrent SCAD–TTS; and (3) scenario-dependent management guidance that accounts for the causal sequence—whether SCAD triggered TTS, TTS triggered SCAD, or both occurred in parallel—with specific recommendations on revascularization strategy, beta-blocker timing, antiplatelet therapy, and hemodynamic support. Conservative management remains the cornerstone for both conditions, but medical therapy and follow-up must be individualized according to the dominant clinical and imaging phenotype. Future multicenter prospective registries with standardized diagnostic protocols are needed to define the true prevalence, shared genetic susceptibility, and optimal management of concurrent SCAD–TTS.

## Figures and Tables

**Table 2:** Medical therapy considerations for concurrent SCAD–TTS.

Medication	SCAD	TTS	Concurrent SCAD–TTS
Aspirin	Recommended: single agent preferred for conservatively managed patients	Uncertain benefit	Monotherapy reasonable
DAPT	Considered post-stent (12 months); uncertain benefit in conservative management; borderline association with higher recurrence in meta-regression	Not routinely indicated	Individualize; avoid if no stent and bleeding risk present
Beta-blockers	Recommended; associated with reduced recurrence in the Vancouver cohort (HR 0.36, *p* = 0.004); formal evidence awaited from BA-SCAD RCT.	Controversial in the acute phase; may worsen LVOTO. Recent meta-analysis (*n* = 11,167): 28% mortality reduction (OR 0.72) and 29% recurrence reduction long-term. Benefit most evident in patients with hypertension	Consider after acute phase resolution; withhold in early TTS if LVOTO is suspected. Carvedilol preferred unless LVOTO present (then β1-selective, e.g., bisoprolol)
ACE-I/ARB	Recommended if LV dysfunction (EF < 50%)	Recommended; first-line in TTS-associated LV dysfunction. Evidence supports long-term mortality and recurrence benefit	Recommended in both; titrate to hemodynamics; can be weaned once LV function recovers if no other indication
MRA	Not routinely indicated	May be considered in the acute phase with significant LV dysfunction (LVEF < 40%); prescribed in up to 43% of TTS patients in observational series	Consider if LVEF is severely reduced; discontinue after LV recovery
Statins	Not routinely indicated (non-atherosclerotic); no clear benefit	Not routinely indicated	Not indicated unless concurrent atherosclerosis or dyslipidemia
Anticoagulation	If LV thrombus or intraluminal thrombus, discontinue systemic anticoagulation once SCAD is confirmed, unless other indications	If apical akinesis/thrombus, prophylactic anticoagulation may be considered (AHA Class IIb). VKA or DOAC for up to 3 months	If apical akinesis/thrombus, balance bleeding risk from IMH extension against thromboembolic risk
P2Y12 inhibitors (potent)	Avoid ticagrelor/prasugrel; clopidogrel only if needed (intimal tear with prothrombotic risk)	Not indicated	Avoid; use aspirin ± clopidogrel only
Catecholamine inotropes	Standard ACS management for cardiogenic shock	Avoid if LVOTO is present; may worsen obstruction. In the absence of LVOTO, dobutamine may be used cautiously	Avoid if LVOTO; prefer levosimendan or mechanical support. If there is no LVOTO, use with caution and reassess for dynamic obstruction
Levosimendan	Not applicable	May be considered in cardiogenic shock without LVOTO as a non-catecholamine inotrope; may accelerate LV recovery. Prospective data lacking	Consider if cardiogenic shock complicates the TTS component; avoid catecholamine inotropes

Recommendations based on Refs: [1,3,6,50,52,80,84]. Abbreviations: ACE-I/ARB: angiotensin-converting enzyme inhibitors/angiotensin receptor blockers; DAPT: dual antiplatelet therapy; DOAC: direct oral anticoagulant; LVEF: left ventricular ejection fraction; IMH: intramural hematoma; LV: left ventricle; LVOTO: left ventricular outflow tract obstruction; MACE: major adverse cardiovascular events; MRA: mineralocorticoid receptor antagonist; OR: odds ratio; P2Y12: platelet adenosine diphosphate receptor subtype; RCT: randomized controlled trial; SAPT: single antiplatelet therapy; SCAD: spontaneous coronary artery dissection; TTS: Takotsubo syndrome; VKA: vitamin K antagonist.

## Data Availability

Not applicable.
